# Supplementary material for: Nomo1 deficiency causes autism-like behavior in zebrafish
Source: EMBO Rep. 2024 Jan 22;25(2):11. doi: 10.1038/s44319-023-00036-y (PMC10897165; doi:10.1038/s44319-023-00036-y)
Supplement: Supplementary file 6 — Appendix [file 44319_2023_36_MOESM6_ESM.pdf]

# Appendix

## Table of contents

Appendix Figure S1....................2
Appendix Figure S2....................2
Appendix Figure S3....................3
Appendix Figure S4....................3
Appendix Figure S5....................4
Appendix Figure S6....................4
Appendix Figure S7....................4
Appendix Figure S8....................5

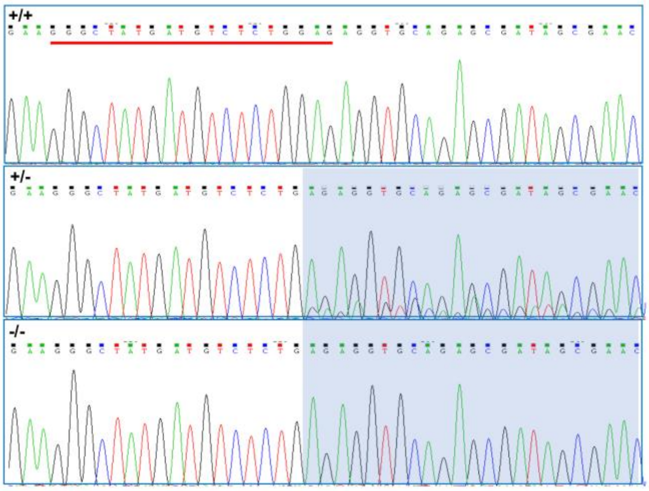


Appendix Figure S1. Alignment of sequences from the successfully generated *nomo1*$^{+/+}$, *nomo1*$^{+/-}$ and *nomo1*$^{-/-}$ zebrafish.

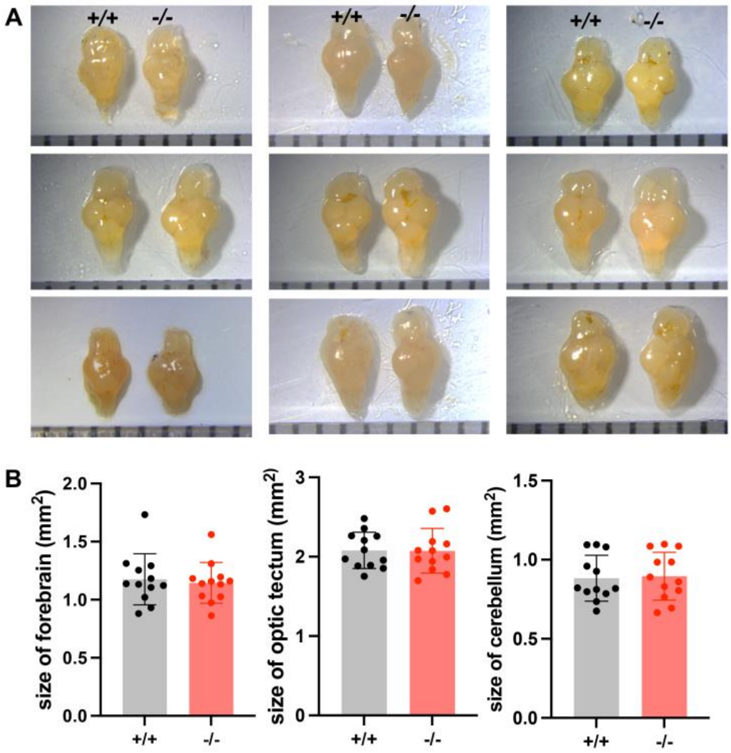


Appendix Figure S2. (A). Whole brain of *nomo1*$^{+/+}$ and *nomo1*$^{-/-}$, the middle square photo also shown in Fig 1D. Ruler attached below each image, cell length 1mm. (B) statistical analysis of forebrain, optic tectum and cerebellum did not show significant differences (biological replicates, N=12, 1 fish in one experiment). Data are analyzed using unpaired t-test and presented as the means ± SEM.

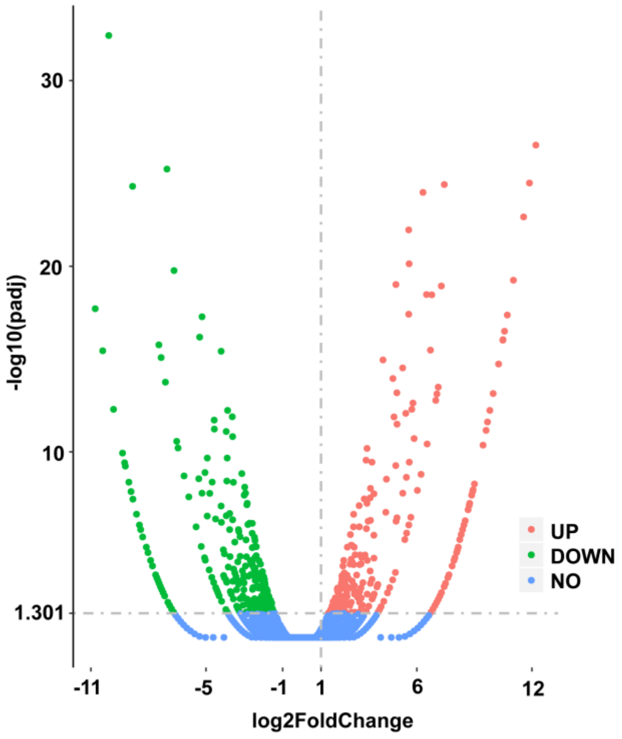


Appendix Figure S3. Volcano map of DEGs between WT and *nomo1*$^{-/-}$ zebrafish. The abscissa and ordinate indicate the fold change in expression of DEGs (log2fold change) and the significance level of the DEGs (-log10padj) between WT and mutant zebrafish, respectively. The upregulated genes are indicated by a red dot, and the downregulated genes are indicated by a green dot. The significant level of enrichment (padj) was set as the multihypothesis test-corrected p-value (p-value< 0.05).

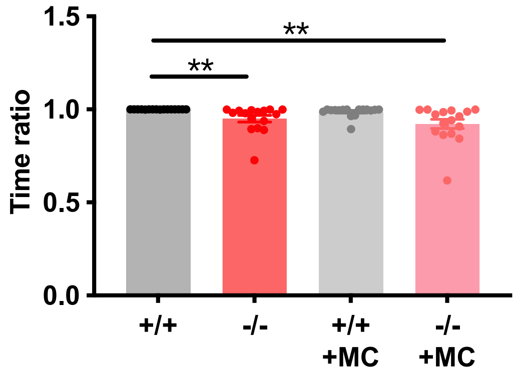


Appendix Figure S4. MC treatment did not increase the swimming time in social area of *nomo1*$^{-/-}$ zebrafish (biological replicates, N=24). Data are analyzed using unpaired t-test and presented as the means ± SEM. $^{**}p < 0.01$

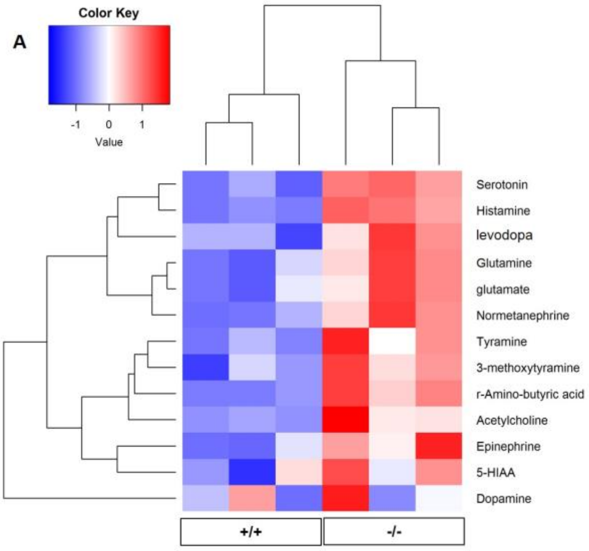


Appendix Figure S5. (A) The clustering analysis of *nomo1*$^{+/+}$ and *nomo1*$^{-/-}$ zebrafish using SRM/MRM revealed that neurotransmitters and metabolites tended to be upregulated in mutant zebrafish.

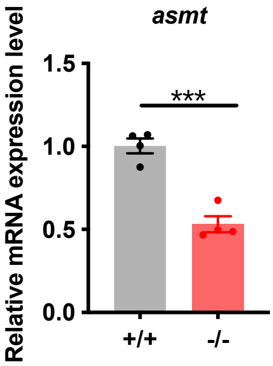


Appendix Figure S6. Relative expression level of *asmt* of *nomo1*$^{+/+}$ and *nomo1*$^{-/-}$ (technical replicates, N=4). Data are analyzed using unpaired t-test and presented as the means ± SEM. $^{***}p < 0.001$

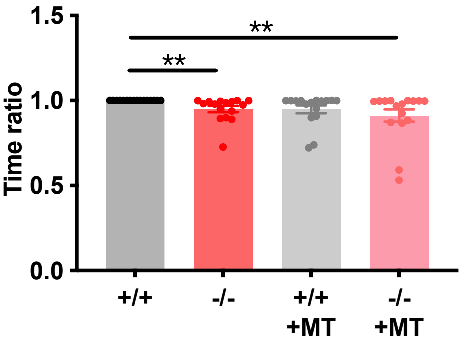


Appendix Figure S7. MT treatment did not increase the swimming time in social area of *nomo1*$^{-/-}$ zebrafish (biological replicates, N=24). Data are analyzed using unpaired t-test and presented as the means ± SEM. $^{**}p < 0.01$

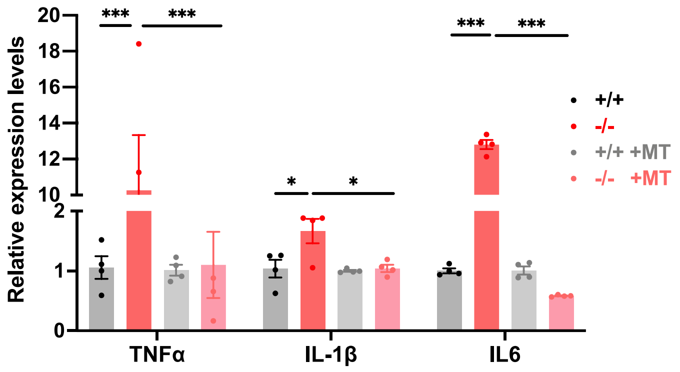


Appendix Figure S8. Relative expression level of cytokines in WT, mutant and MT treatment brains (technical replicates, N=4). Data are analyzed using unpaired t-test and presented as the means ± SEM. $^{*}p < 0.1$, $^{***}p < 0.001$
