# Supplementary figures and images for: Nomo1 deficiency causes autism-like behavior in zebrafish
Source: EMBO Rep. 2024 Jan 22;25(2):11. doi: 10.1038/s44319-023-00036-y (PMC10897165; doi:10.1038/s44319-023-00036-y)

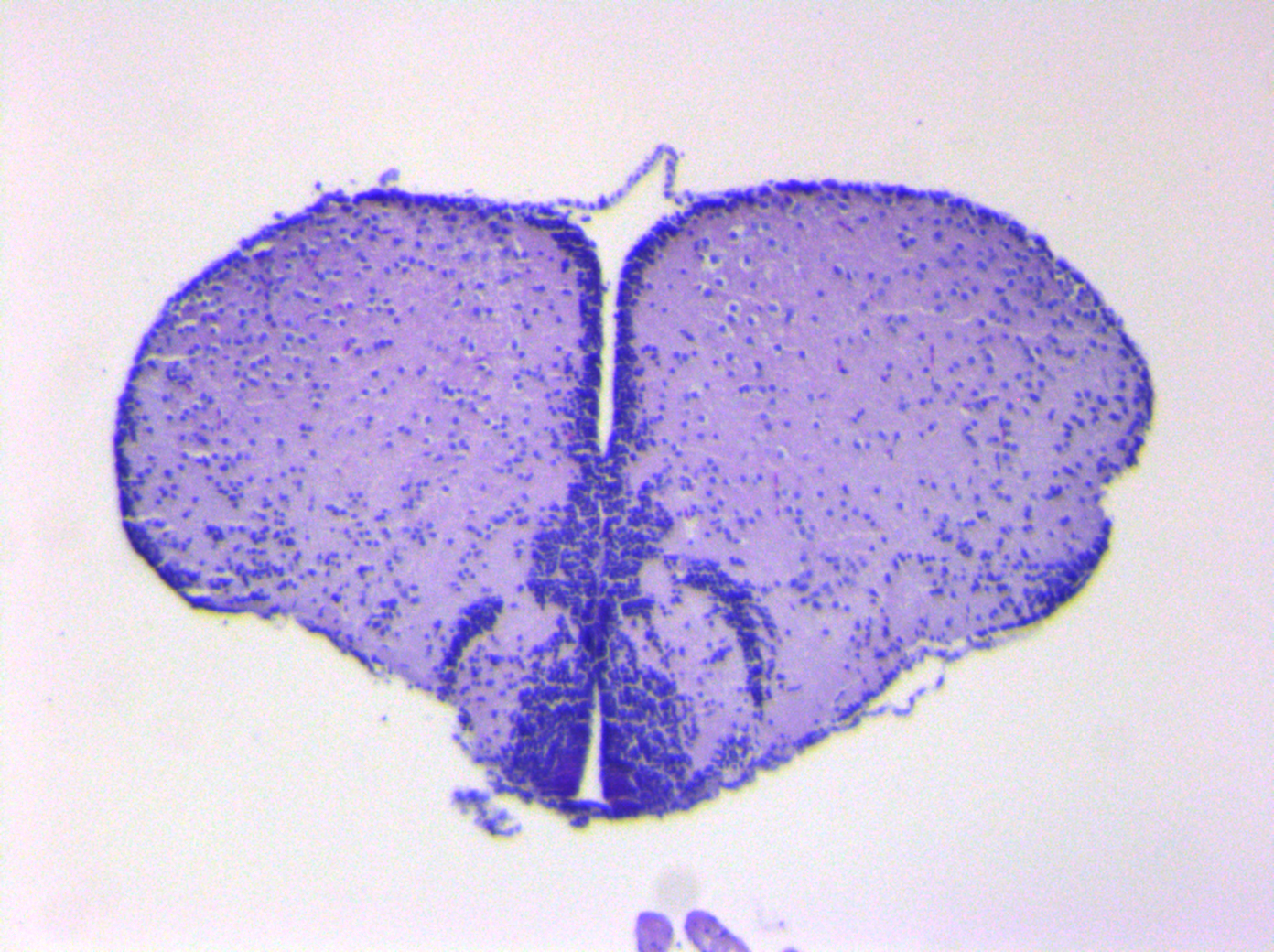

Supplement: Supplementary file 14 — Source Data Fig. 1 [file 44319_2023_36_MOESM14_ESM.zip › Figure 1/1F/+:+ forebrain 3mpf.tif]

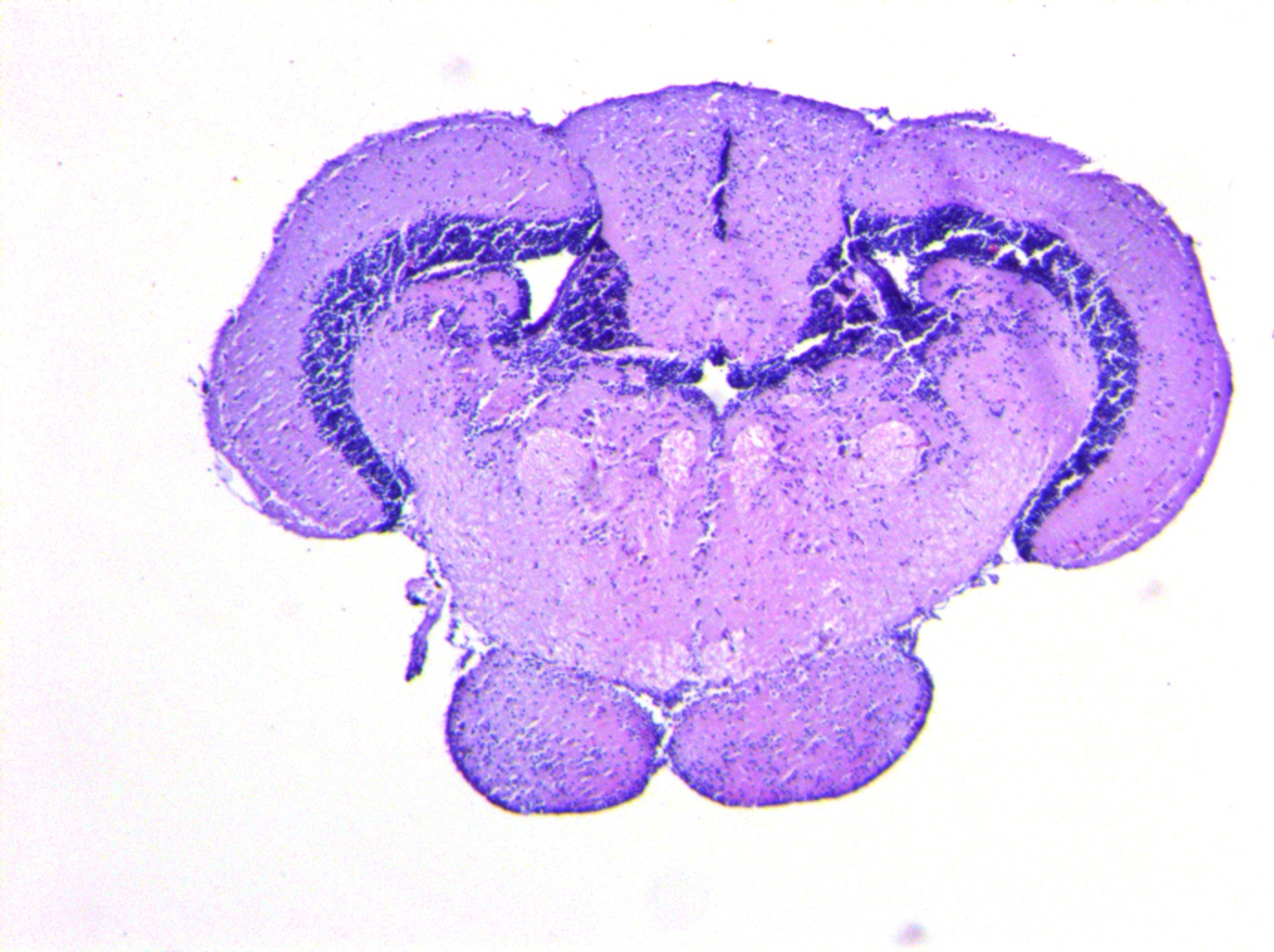

Supplement: Supplementary file 14 — Source Data Fig. 1 [file 44319_2023_36_MOESM14_ESM.zip › Figure 1/1F/+:+ hindbrain 3mpf.tif]

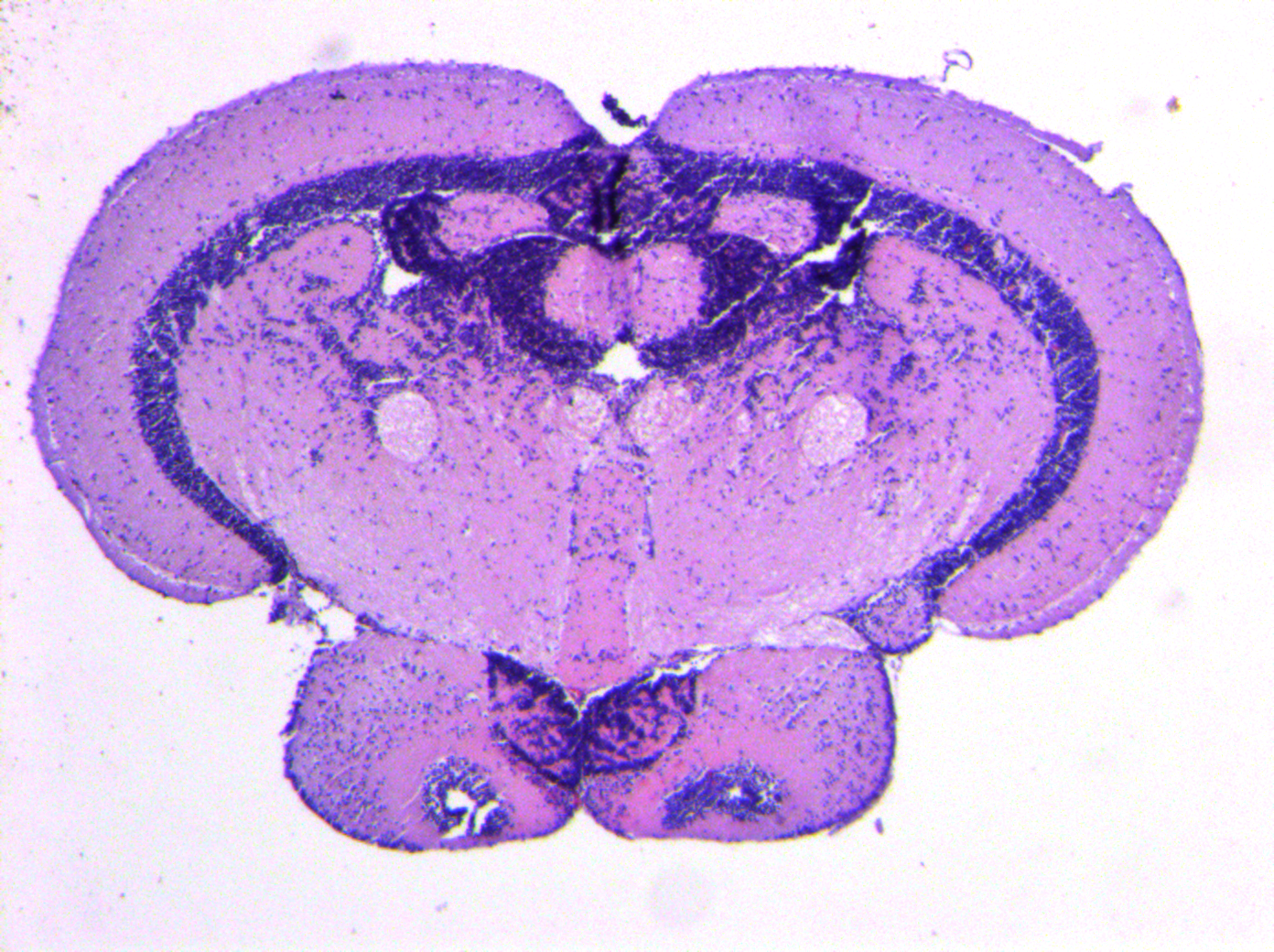

Supplement: Supplementary file 14 — Source Data Fig. 1 [file 44319_2023_36_MOESM14_ESM.zip › Figure 1/1F/+:+ midbrain 3mpf.tif]

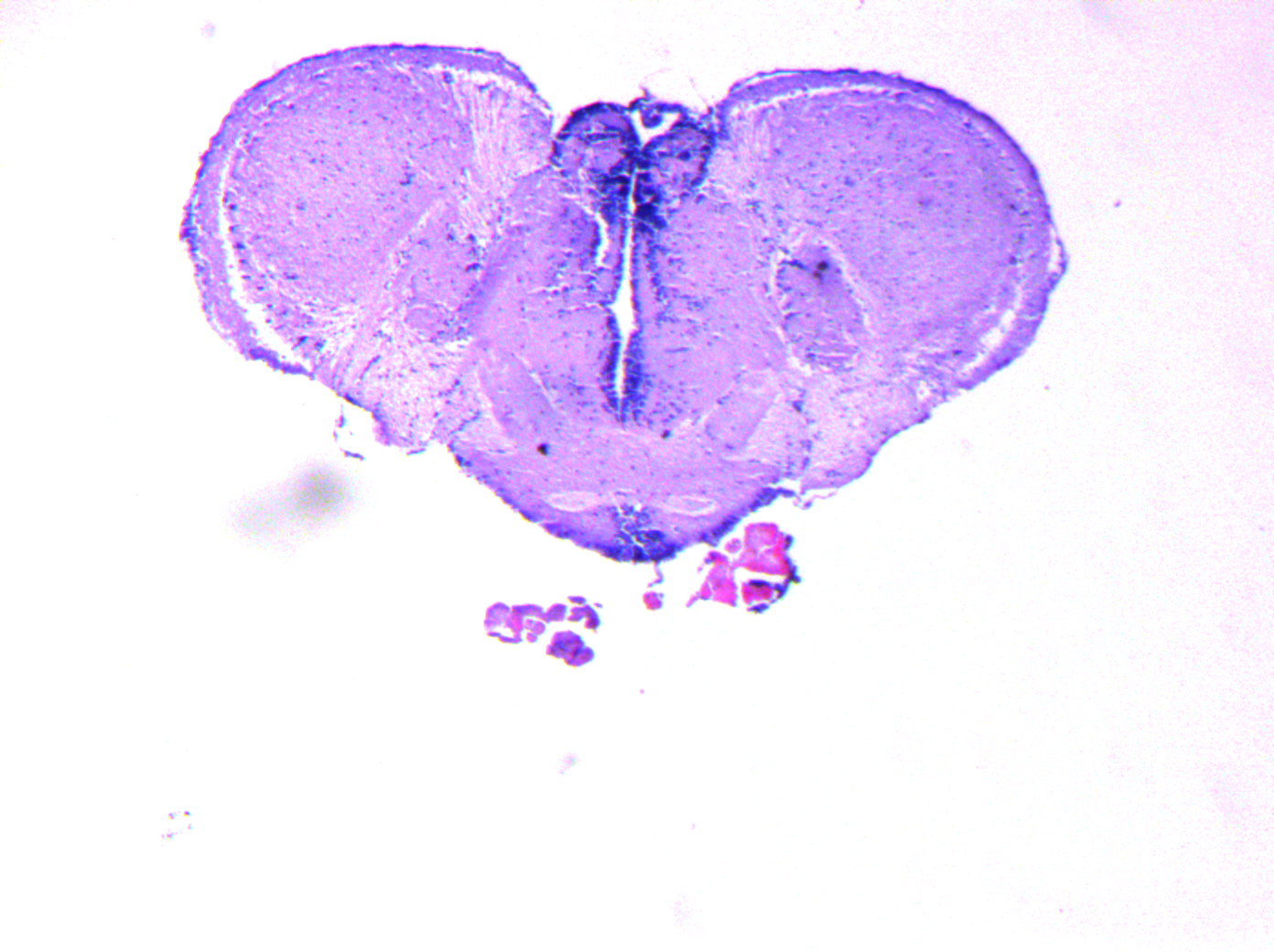

Supplement: Supplementary file 14 — Source Data Fig. 1 [file 44319_2023_36_MOESM14_ESM.zip › Figure 1/1F/+:+ telencephalon 3mpf.tif]

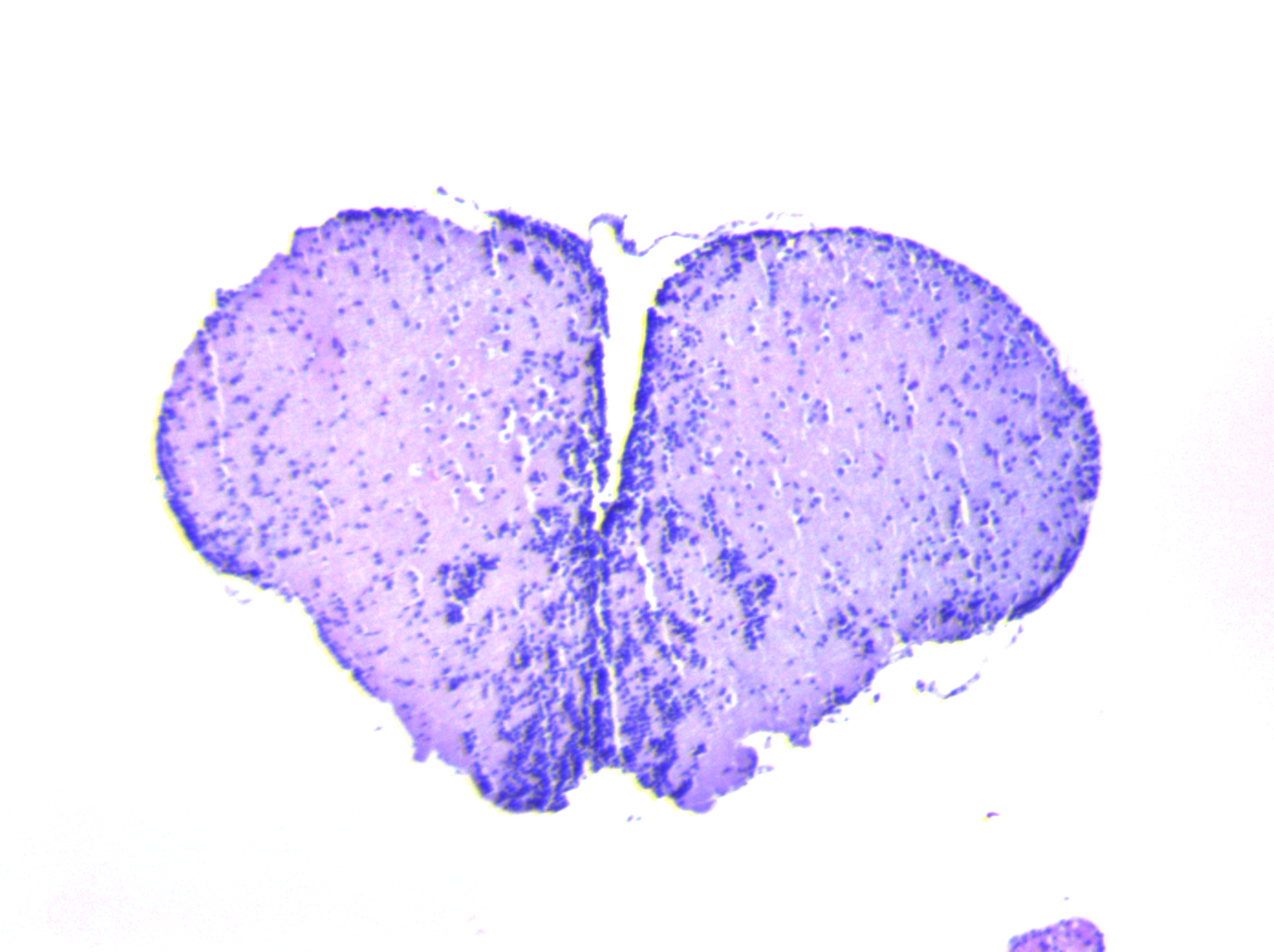

Supplement: Supplementary file 14 — Source Data Fig. 1 [file 44319_2023_36_MOESM14_ESM.zip › Figure 1/1F/-:- forebrain 3mpf.tif]

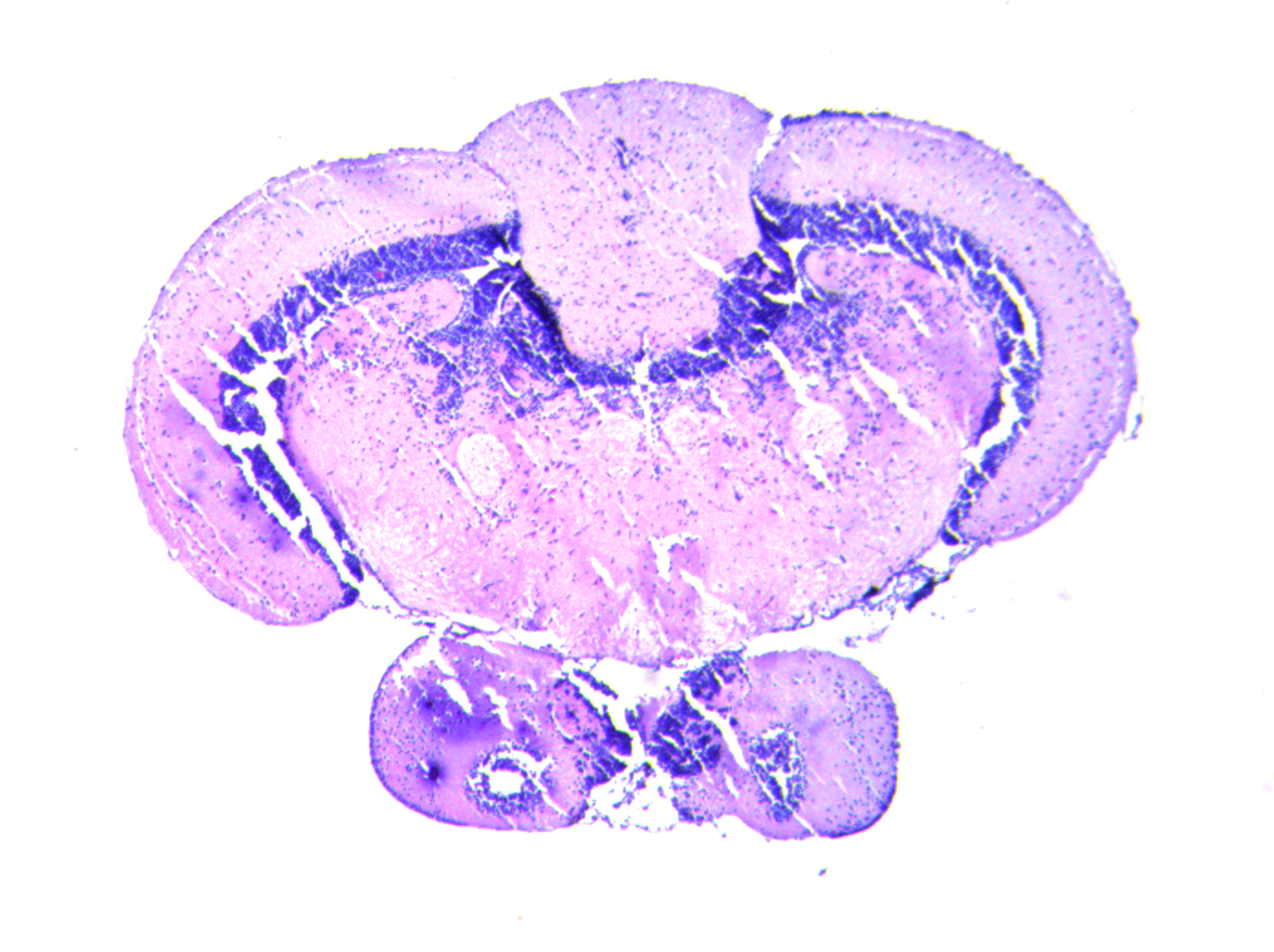

Supplement: Supplementary file 14 — Source Data Fig. 1 [file 44319_2023_36_MOESM14_ESM.zip › Figure 1/1F/-:- hindbrain 3mpf.tif]

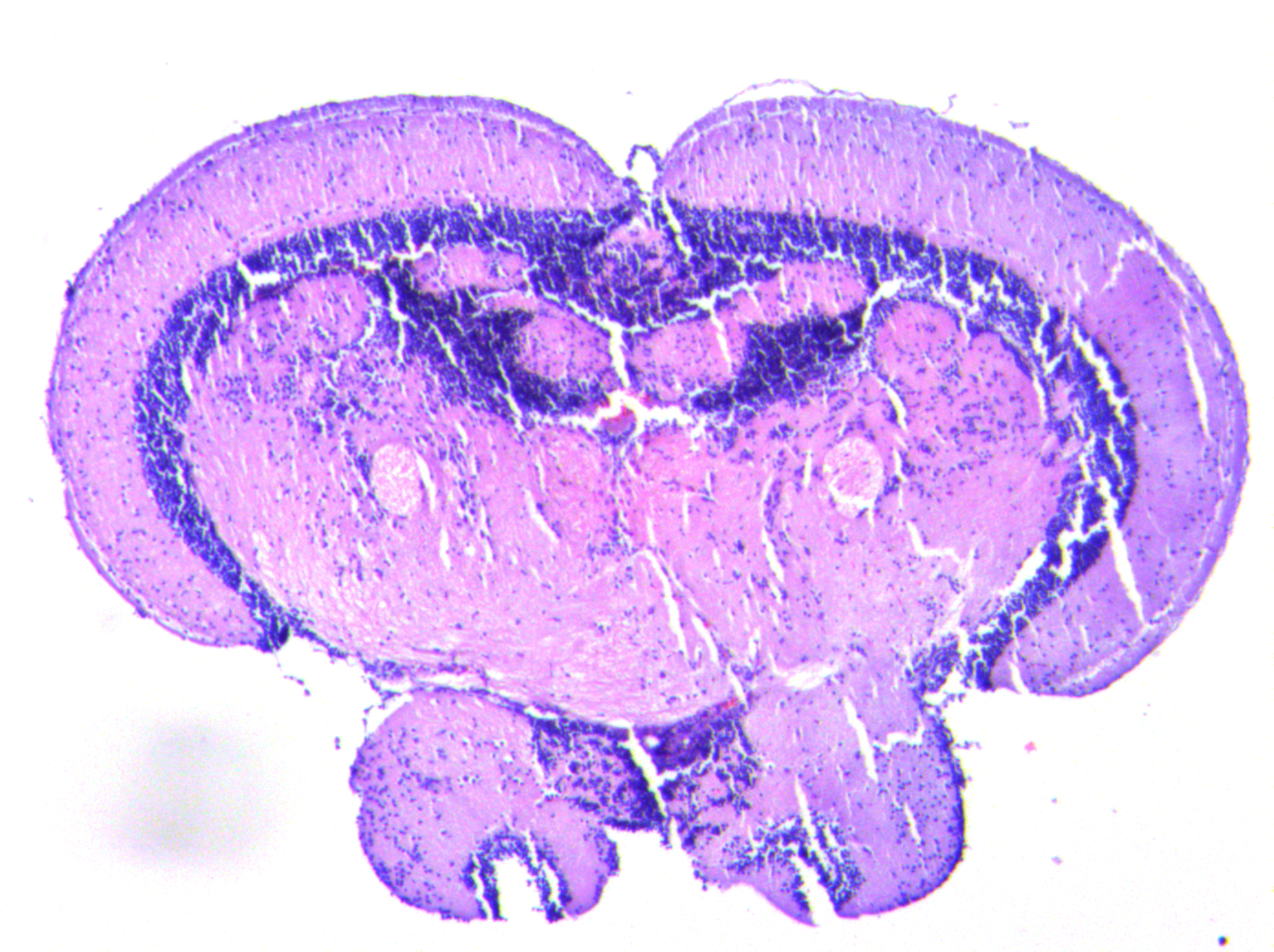

Supplement: Supplementary file 14 — Source Data Fig. 1 [file 44319_2023_36_MOESM14_ESM.zip › Figure 1/1F/-:- midbrain 3mpf.tif]

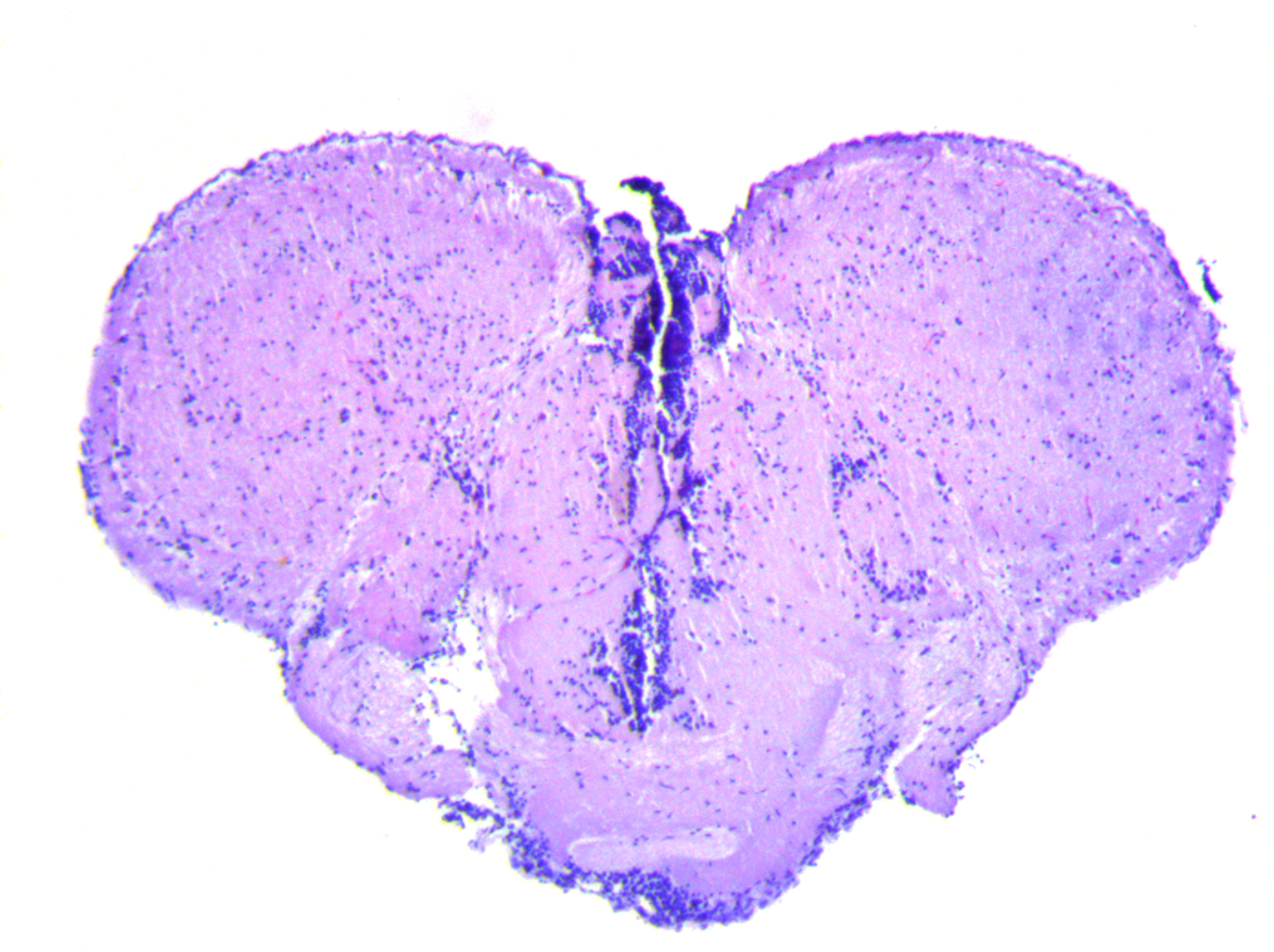

Supplement: Supplementary file 14 — Source Data Fig. 1 [file 44319_2023_36_MOESM14_ESM.zip › Figure 1/1F/-:- telencephalon 3mpf.tif]

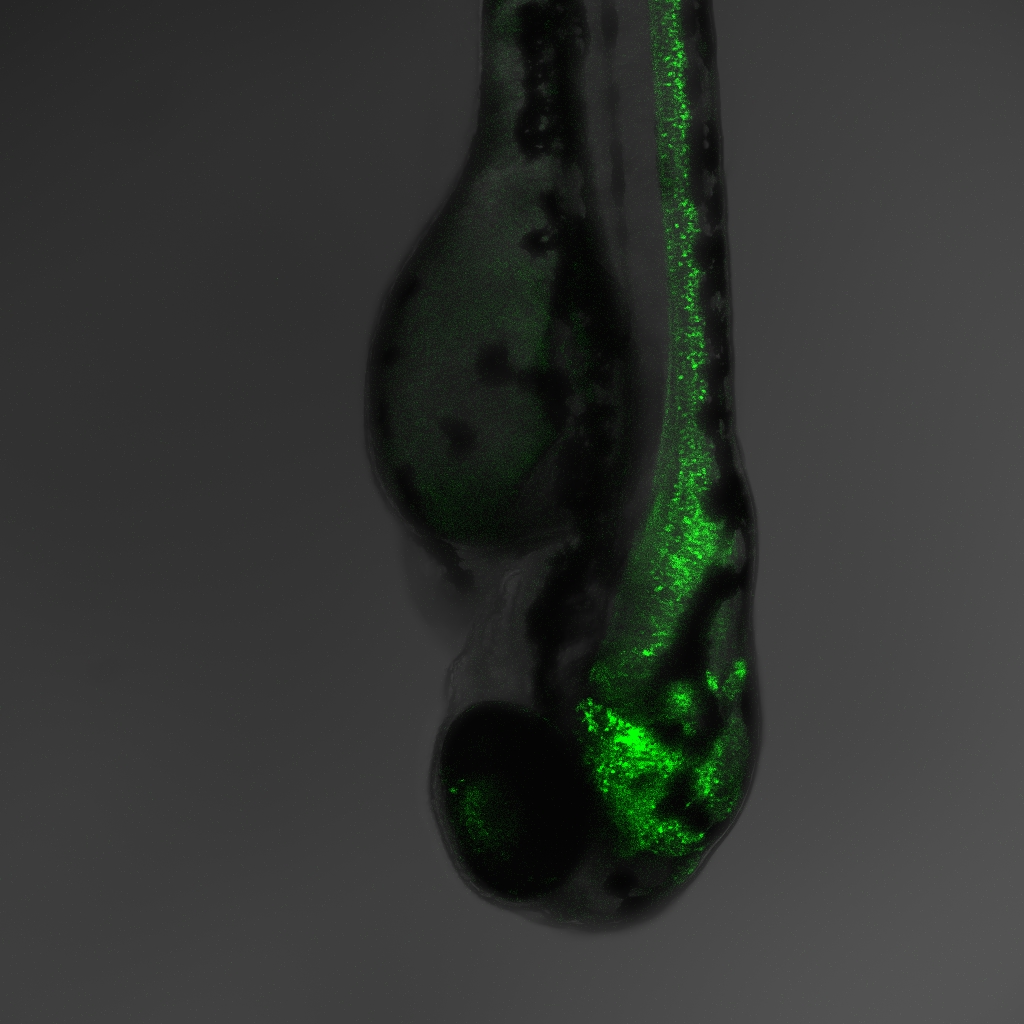

Supplement: Supplementary file 18 — Source Data Fig. 5 [file 44319_2023_36_MOESM18_ESM.zip › Figure 5/5A/+:+ 3dpf.jpg]

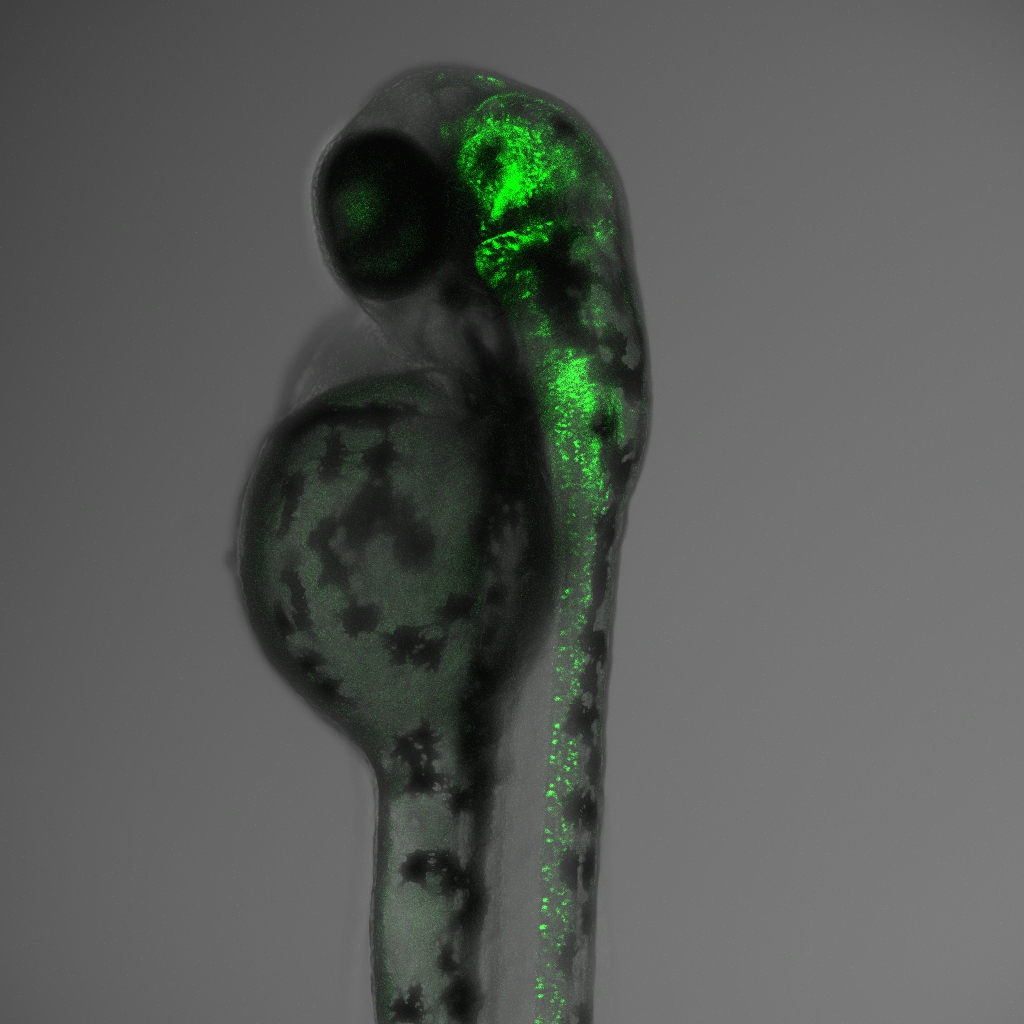

Supplement: Supplementary file 18 — Source Data Fig. 5 [file 44319_2023_36_MOESM18_ESM.zip › Figure 5/5A/+:+ 48hpf.jpg]

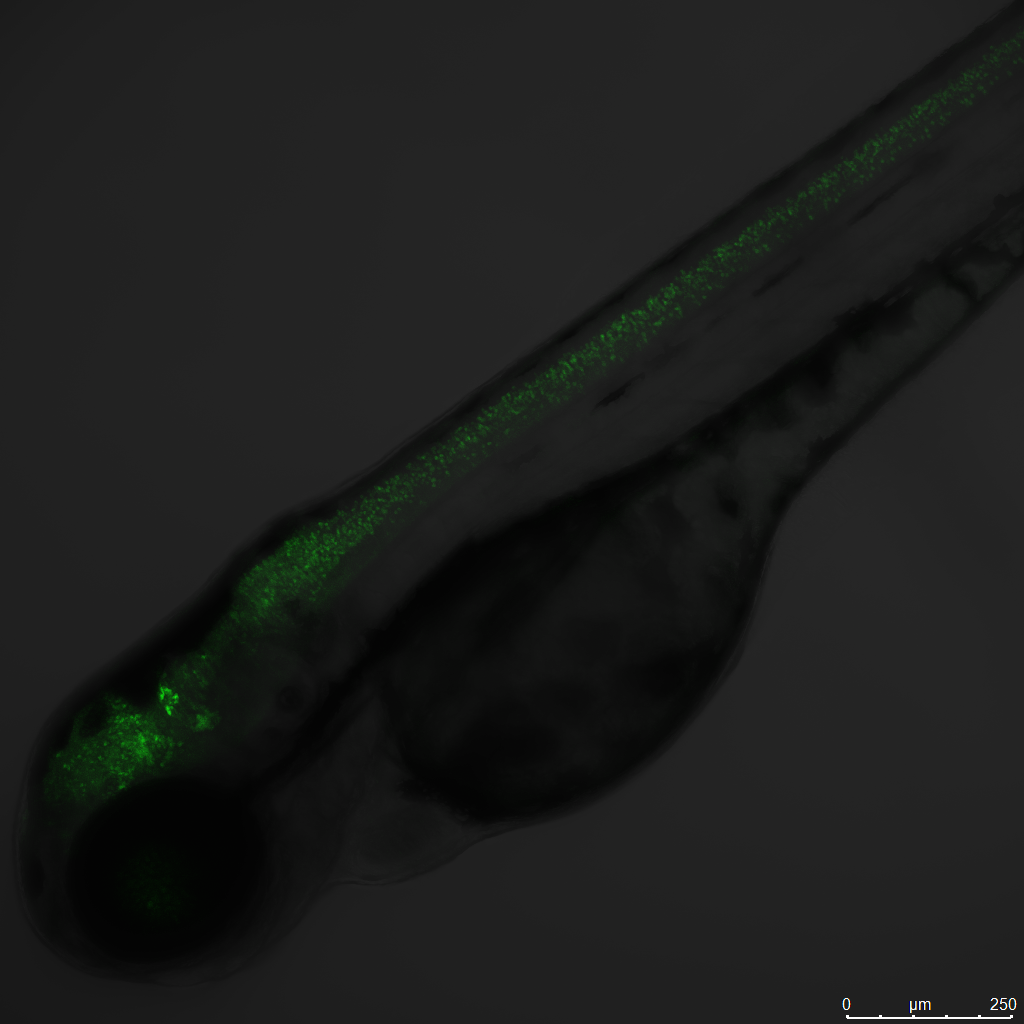

Supplement: Supplementary file 18 — Source Data Fig. 5 [file 44319_2023_36_MOESM18_ESM.zip › Figure 5/5A/-:- 3dpf.tif]

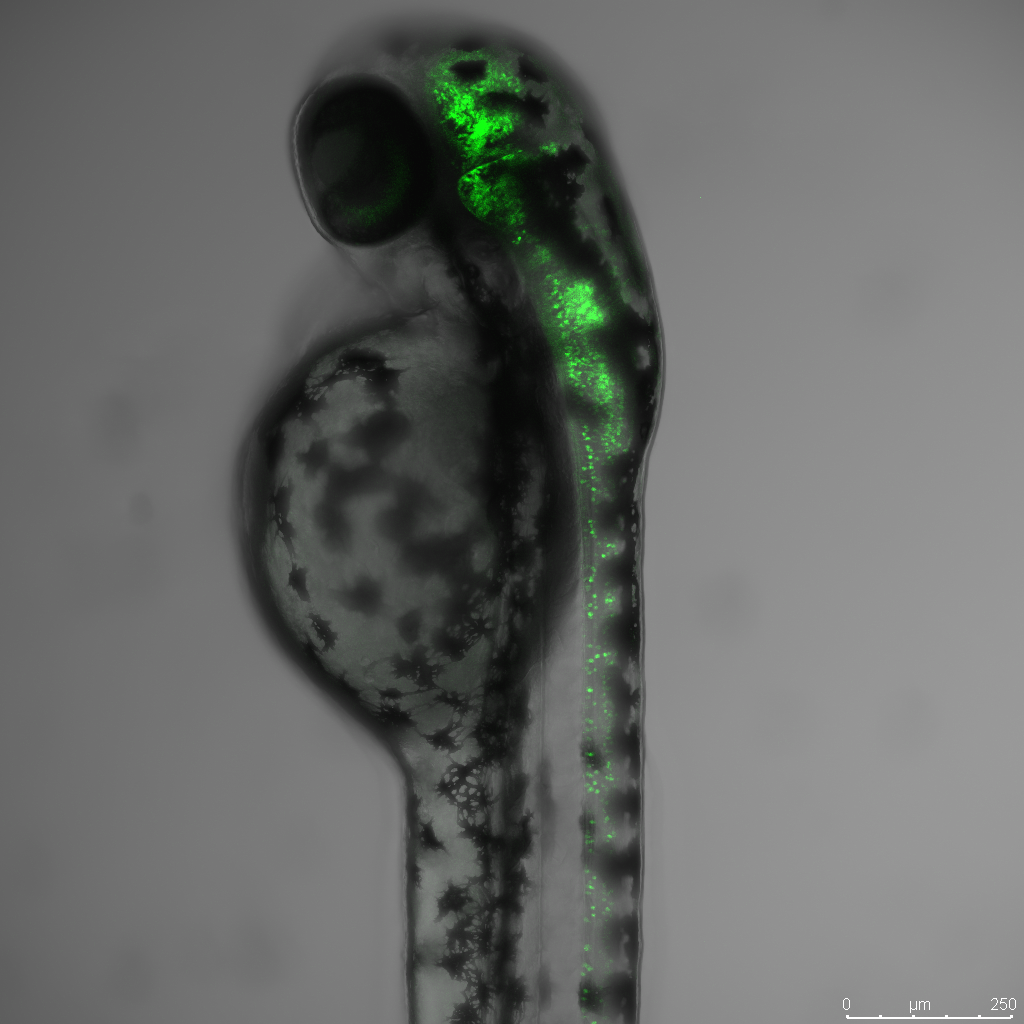

Supplement: Supplementary file 18 — Source Data Fig. 5 [file 44319_2023_36_MOESM18_ESM.zip › Figure 5/5A/-:- 48hpf.tif]

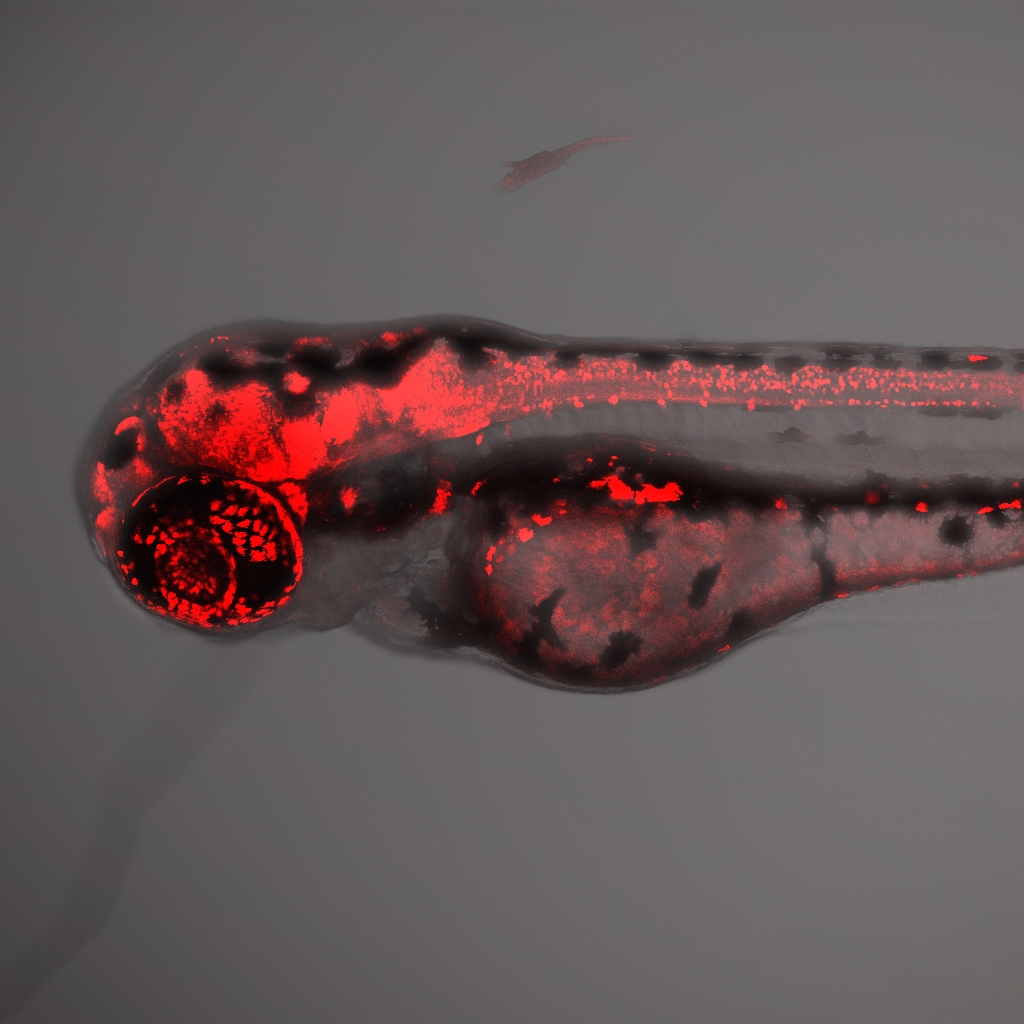

Supplement: Supplementary file 18 — Source Data Fig. 5 [file 44319_2023_36_MOESM18_ESM.zip › Figure 5/5B/+:+ 3dpf.jpg]

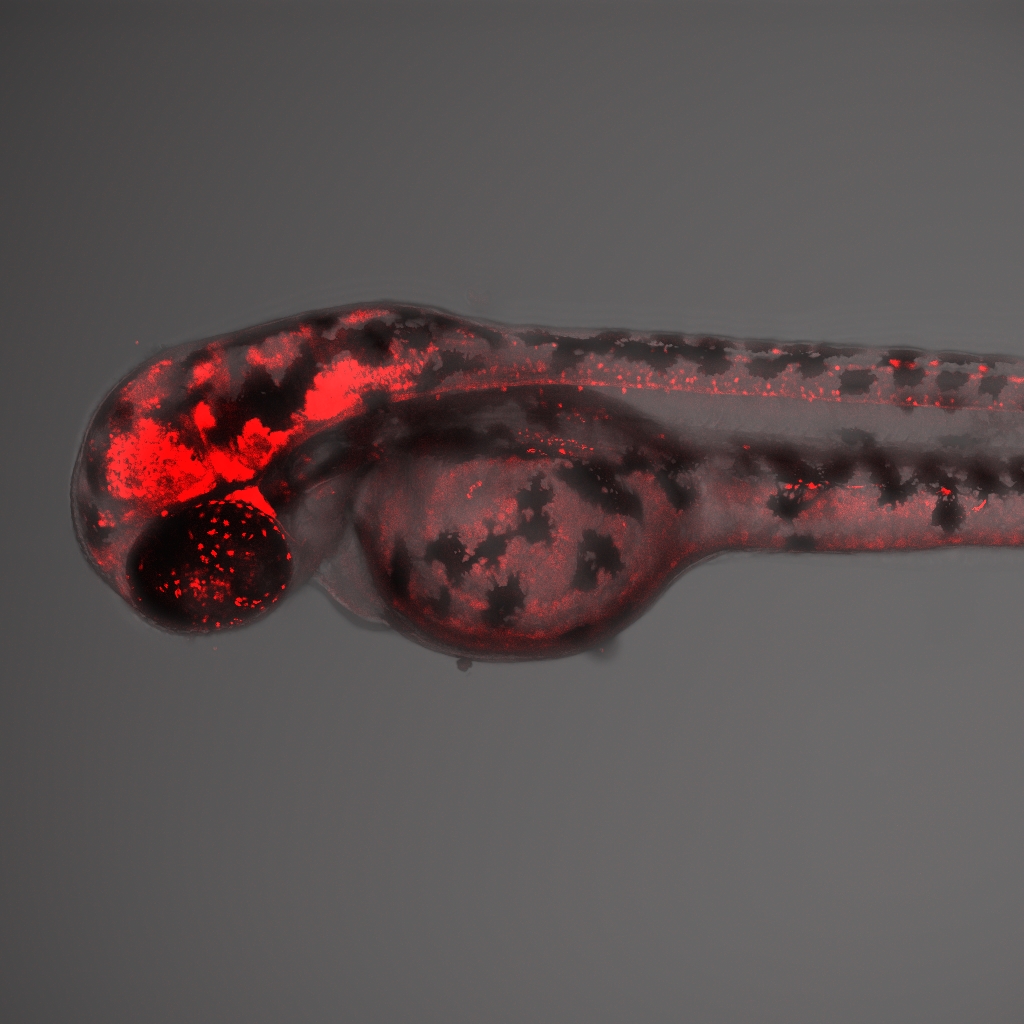

Supplement: Supplementary file 18 — Source Data Fig. 5 [file 44319_2023_36_MOESM18_ESM.zip › Figure 5/5B/+:+ 48hpf.jpg]

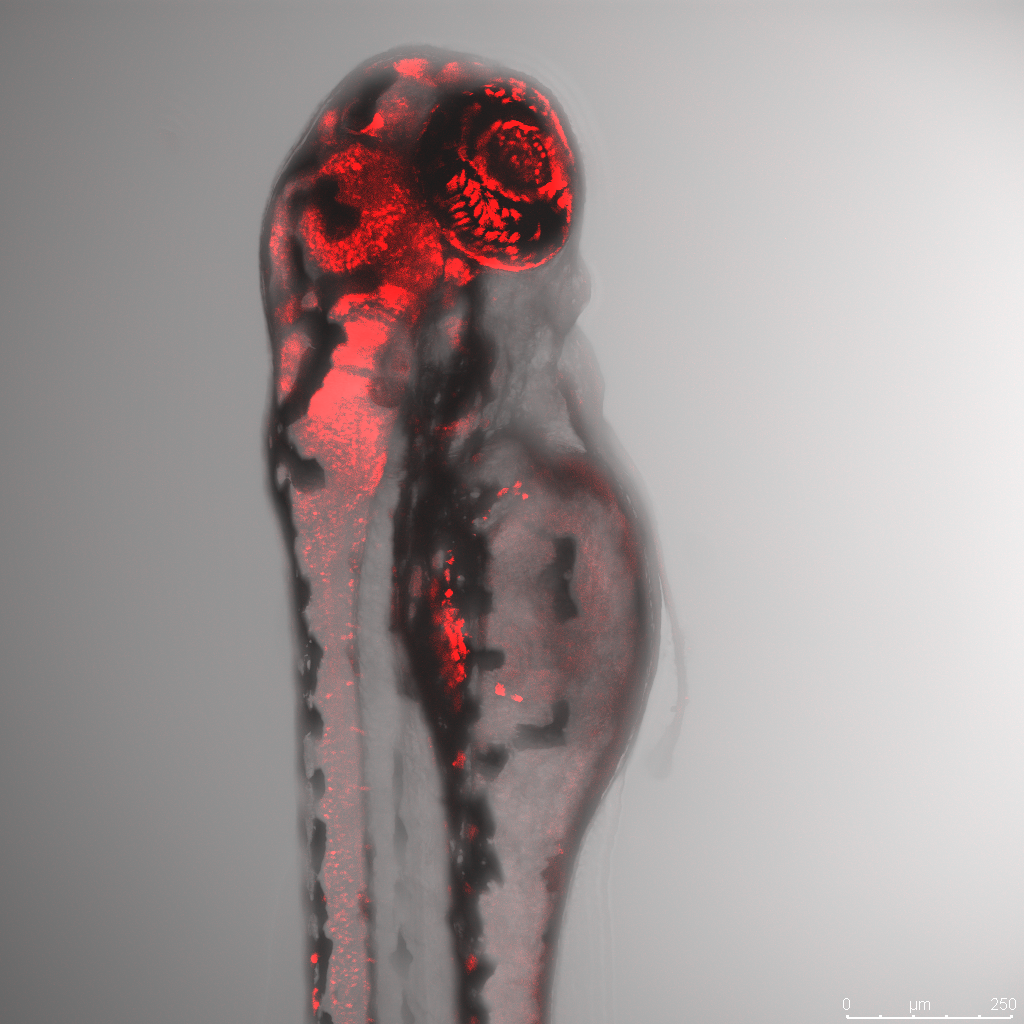

Supplement: Supplementary file 18 — Source Data Fig. 5 [file 44319_2023_36_MOESM18_ESM.zip › Figure 5/5B/-:- 3dpf.tif]

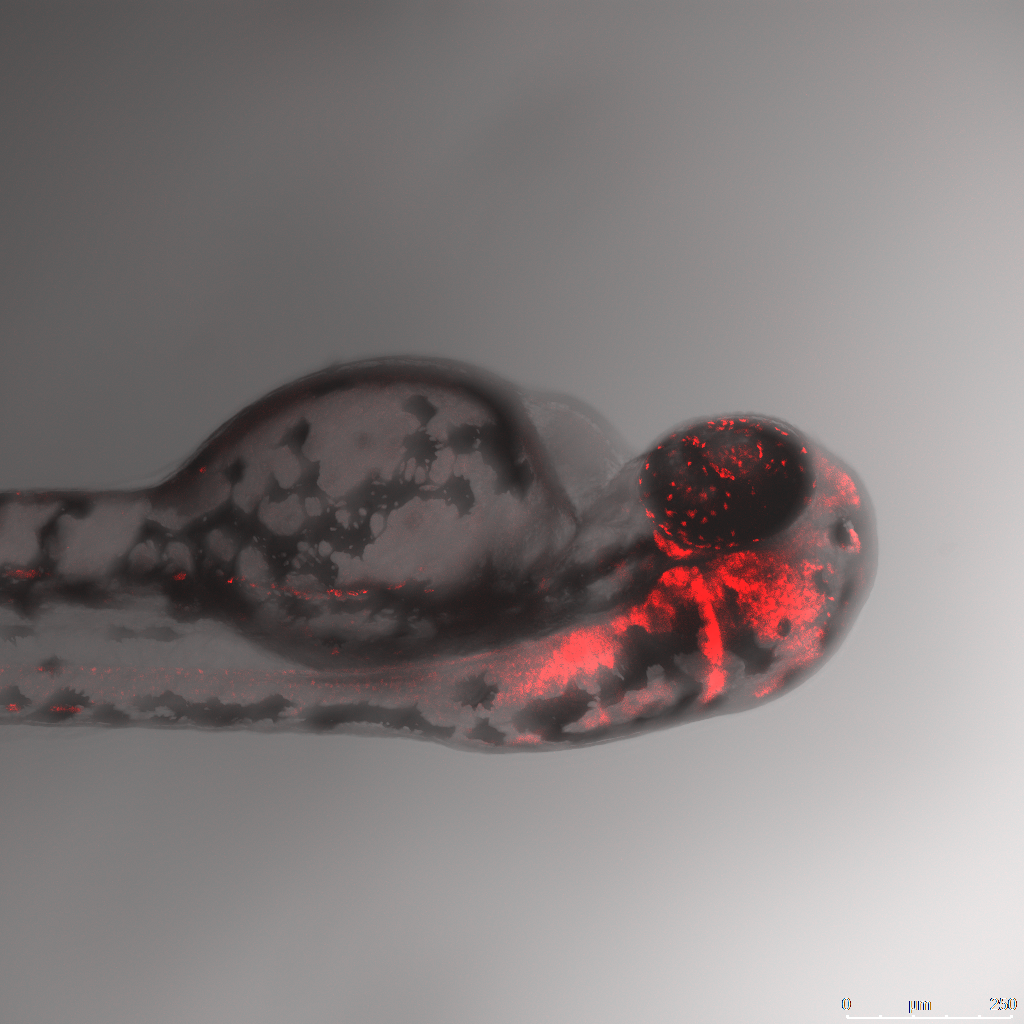

Supplement: Supplementary file 18 — Source Data Fig. 5 [file 44319_2023_36_MOESM18_ESM.zip › Figure 5/5B/-:- 48hpf.tif]

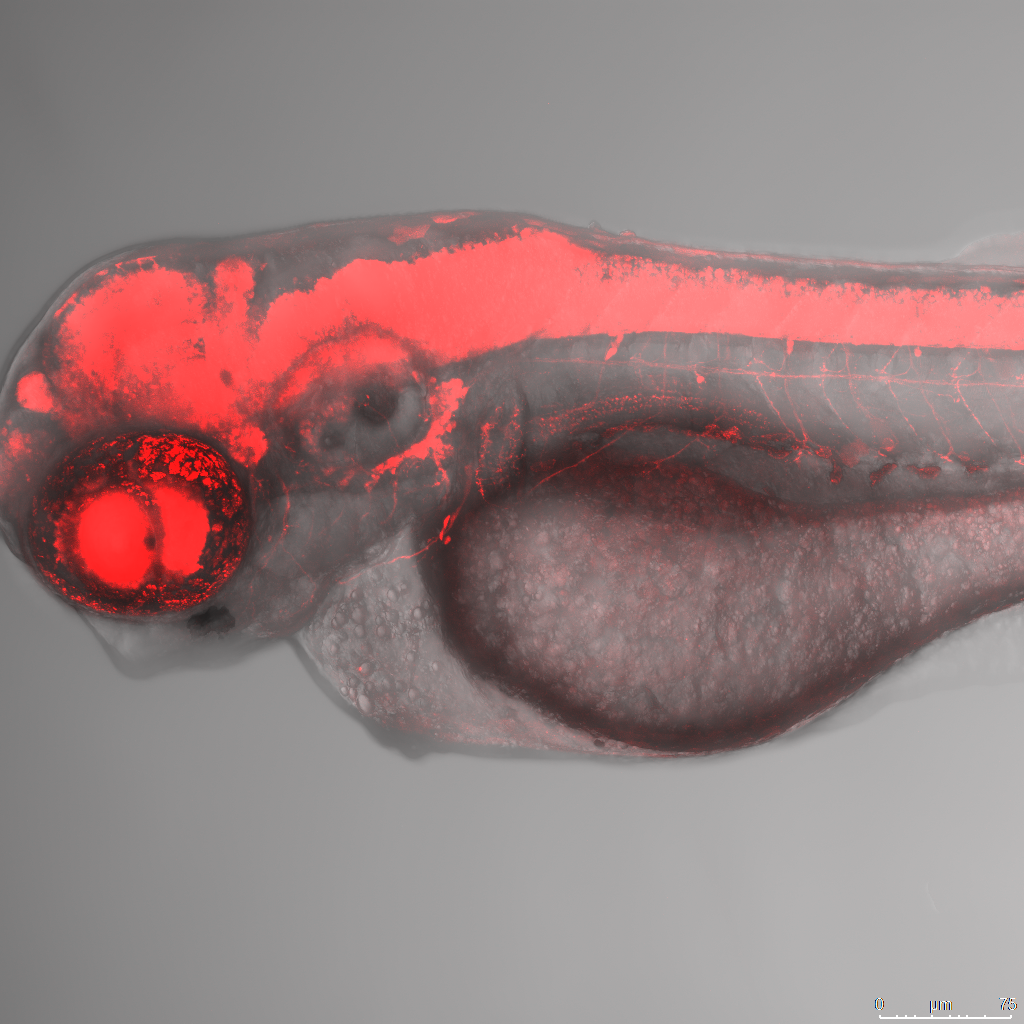

Supplement: Supplementary file 18 — Source Data Fig. 5 [file 44319_2023_36_MOESM18_ESM.zip › Figure 5/5C/+:+ 3dpf.tif]

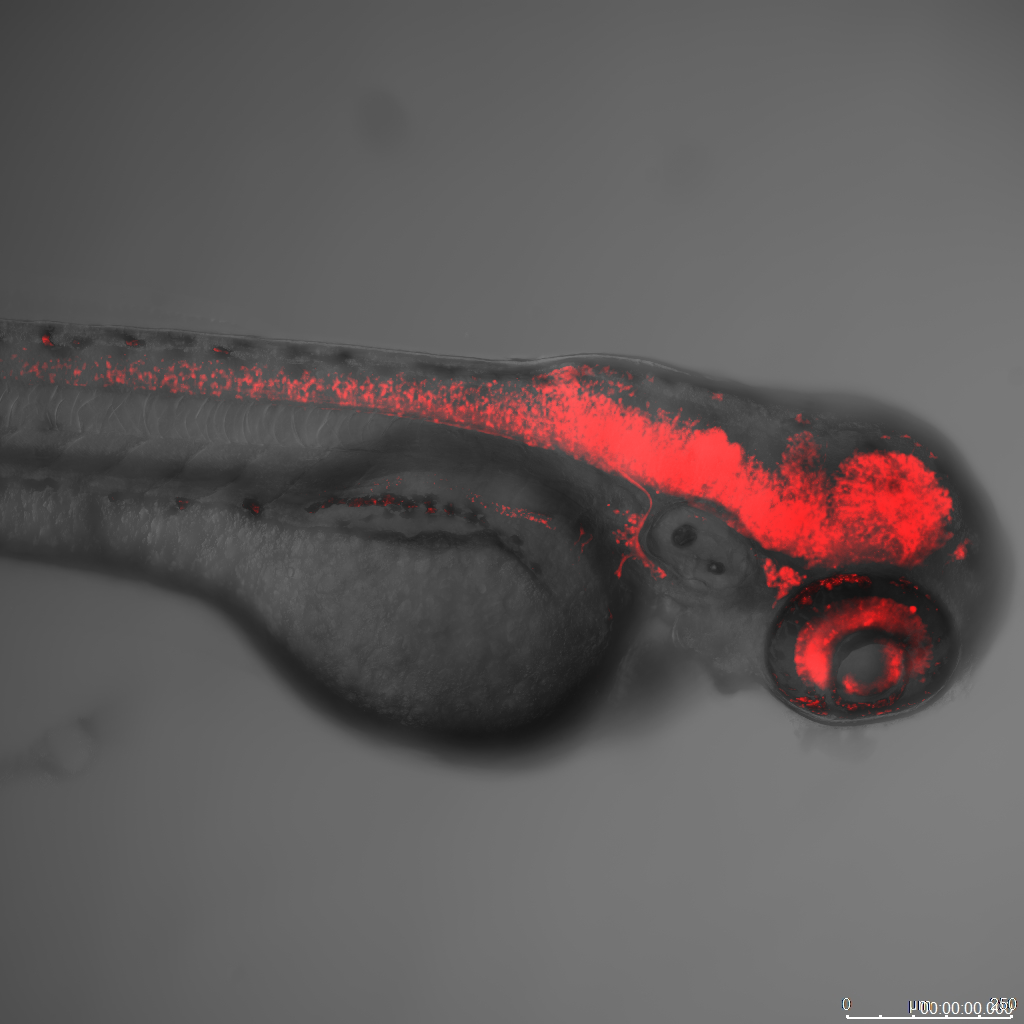

Supplement: Supplementary file 18 — Source Data Fig. 5 [file 44319_2023_36_MOESM18_ESM.zip › Figure 5/5C/-:- 3dpf.tif]

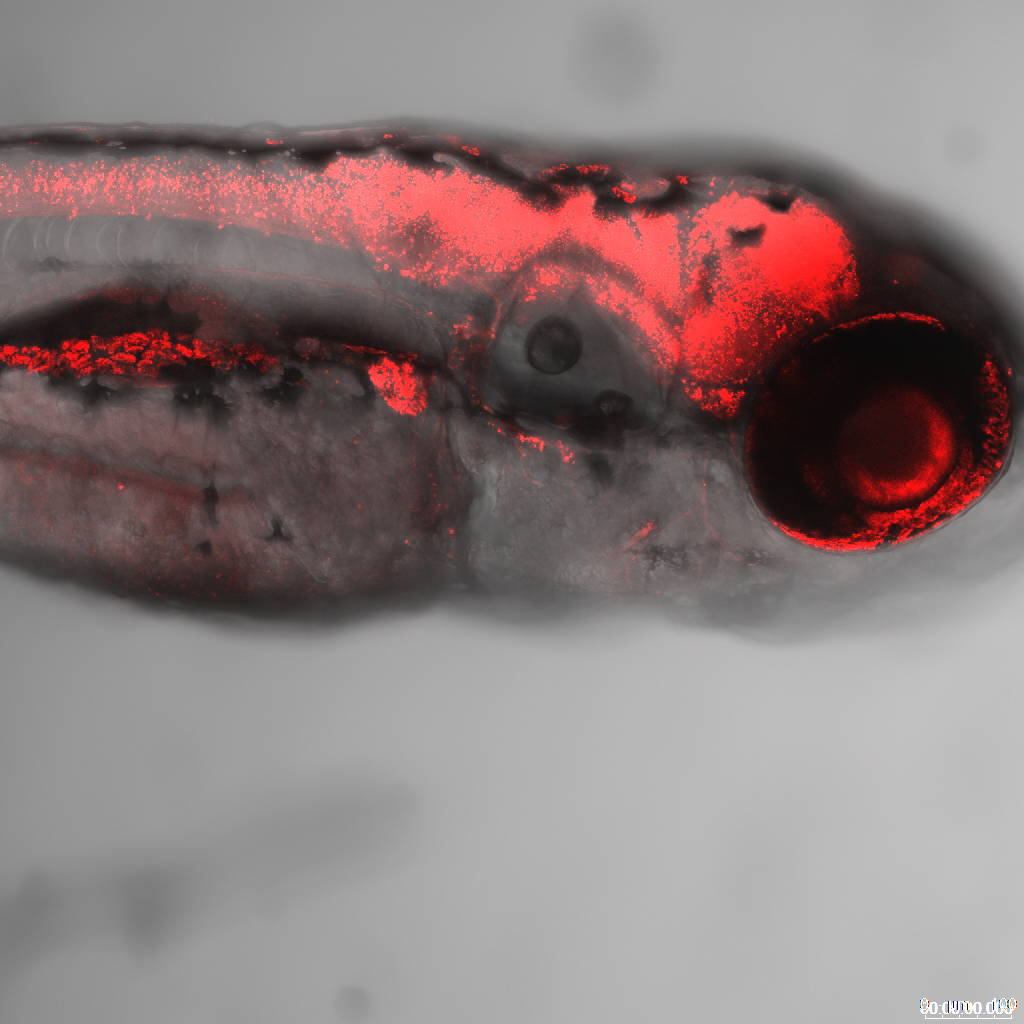

Supplement: Supplementary file 18 — Source Data Fig. 5 [file 44319_2023_36_MOESM18_ESM.zip › Figure 5/5D/+:+ 6dpf.png]

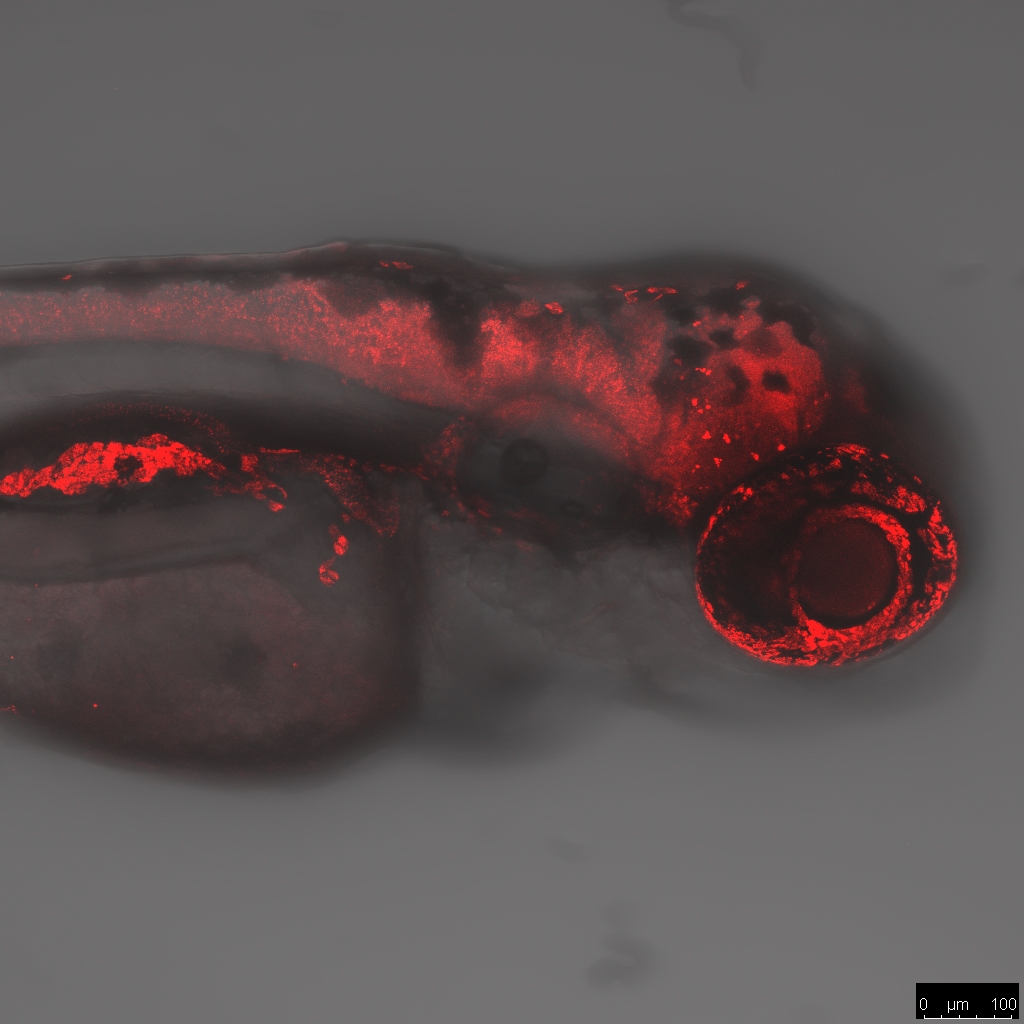

Supplement: Supplementary file 18 — Source Data Fig. 5 [file 44319_2023_36_MOESM18_ESM.zip › Figure 5/5D/-:- 6dpf.jpg]
