# Supplementary material for: Nomo1 deficiency causes autism-like behavior in zebrafish
Source: EMBO Rep. 2024 Jan 22;25(2):11. doi: 10.1038/s44319-023-00036-y (PMC10897165; doi:10.1038/s44319-023-00036-y)
Supplement: Supplementary file 22 — Expanded View Figures [file 44319_2023_36_MOESM22_ESM.pdf]

Expanded View Figures

Figure EV1. Protein sequence of zebrafish WT and mutant Nomo1 protein.

(**A**, **B**) Amino acid sequences of WT and mutant Nomo1 protein.

© The Author(s)

A WT Nomo1 protein sequence

MYVSARLQCCTMGGIKELAILTLSFLYFTFVNATDDILVGCGGFVKSDVEINYSVIEIKLYTKQGSLKYQTDCAPINGYFMIPLY
1 10 20 30 40 50 60 70 80

DKGDFVLKIEPPSGWSFEPTTVDLHVDGVTDICTKEQDINFVFTGFSVLGTVLSKGHLLGPAGVEVSLRKAGEDAVLQSVFTHAG
90 100 110 120 130 140 150 160 170

GQYTFLKVLPGSYDITASHSSWTLEQSSTAVVVSNENAPAAAPLVVKGYDVSGEVQSDSEPMKGVSFLLYSASVTKEDISGCAVA
180 190 200 210 220 230 240 250

PVDGALVGDASLVYLCSSQSREDGTFSFPCLPSGEYTVVPYYRGERITFDVAPSRMDFKVEHSSLTLQPVFRVMGFSVMGRVLNG
260 270 280 290 300 310 320 330 340

PDGEGVADAVVTLNNQIKVETKEDGSFRLENMTTGTYTINTHKELMFFEPVTVKIAPSTPQLPDIITAGFSVCGHISVTRLPETV
350 360 370 380 390 400 410 420

KQLGRYKVTLSAQRQDQGFFRTVESDSHGAFCFQVKPGDYSVQVTLPESEVKAGLALQPHSLDISLVDRPVTDLLFTQFIASVSG
430 440 450 460 470 480 490 500 510

SVSCLVACGDLTVSLQPVSRQGERQNFQLSGSSETLTFTFGNVLPGKYKVSITQEEWCWKHKSVEIDVLDSYVEGVEFRQTGYLL
520 530 540 550 560 570 580 590

RCSLSHAITLEFFQDGSLPENVGVYNLSKGVNRFCLSKPGVYKVTPRSCHQFEQDYYTYNTSAPSILTLTAVRHHMTGLITTDKM
600 610 620 630 640 650 660 670 680

LDVTVTIKSSIESEPALVLGPLRSNEEQRREQQLLEIAARKKERGEAGDEKSPPVEEKPEELREPFHYEFSYWARAGEKITVTPS
690 700 710 720 730 740 750 760

SKEFLFYPPEVEATITGENCPGRLVEITGRAGLFLTGQVAPTLEGVEITIKESKATTPLITVLTDENGAYSVGPLHSDSQYDISA
770 780 790 800 810 820 830 840 850

SKEGFVLTPVEGKTGDFKAFALAGVTFEIKAEDGVPLSGVLLSLSGASFRSNLLTQDTGLLTFNNLSPGQYYFKPMMKEFRFEPS
860 870 880 890 900 910 920 930

AQMITVEEGQVLHIPITGFKTAYSCYGTVQSIGGDAEQGVAVEAVGQSECGMYSEDTVTDEEGRFRLRGLRPGCNYNIQLRGEGN
940 950 960 970 980 990 1000 1010 1020

DHIERALPPHKTIEVGNTDIDGINIIAFRQINQFDLSGNVITSPEHLPTLWVKLYKSDNLDNPFQSVSLGQSLFFHFQPLPRDGE
1030 1040 1050 1060 1070 1080 1090 1100

SYVLMLDTSLSRSQYDFKLPQVSFTSSGYHKHVTLTFNPKRKIPDQDVAQGSFIALPLTLLLLLAVYNHERVIPLLLQLVSQIQG
1110 1120 1130 1140 1150 1160 1170 1180 1190

VRGLAQASGDGLPVDEAKRPSKRPKTRRT*
1200 1210 1220

B mutant Nomo1 protein sequence

MYVSARLQCCTMGGIKELAILTLSFLYFTFVNATDDILVGCGGFVKSDVEINYSVIEIKLYTKQGSLKYQTDCAPINGYFMIPLY
1 10 20 30 40 50 60 70 80

DKGDFVLKIEPPSGWSFEPTTVDLHVDGVTDICTKEQDINFVFTGFSVLGTVLSKGHLLGPAGVEVSLRKAGEDAVLQSVFTHAG
90 100 110 120 130 140 150 160 170

GQYTFLKVLPGSYDITASHSSWTLEQSSTAVVVSNENAPAAAPLVVKGYDVSERCRAIANP*
180 190 200 210 220 230 232

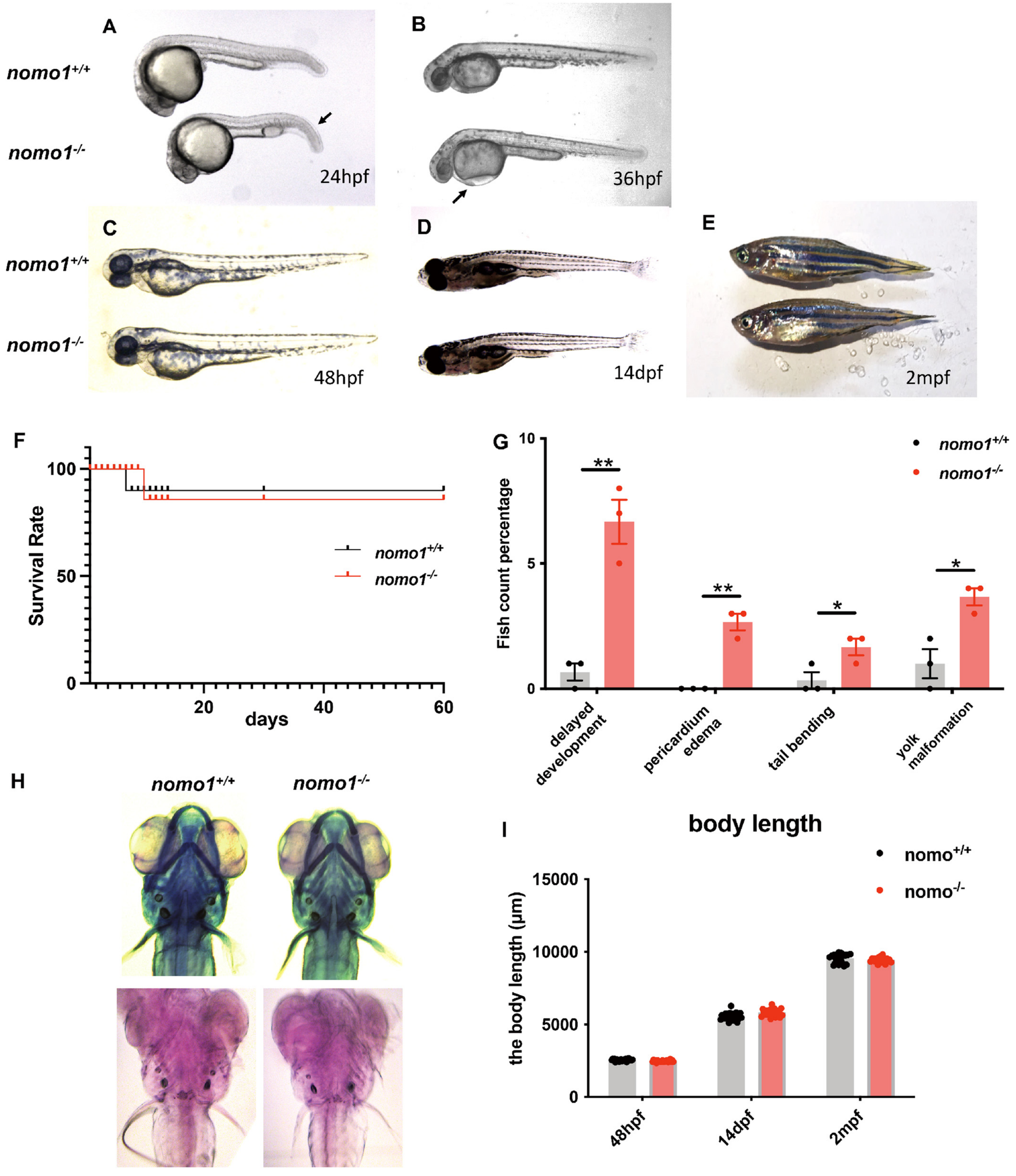
A
B
nomo1+/+
nomo1-/-
24hpf
36hpf
C
D
E
nomo1+/+
nomo1-/-
48hpf
14dpf
2mpf
F
Survival Rate
100
50
0
20
40
60
days
nomo1+/+
nomo1-/-
G
Fish count percentage
10
5
0
**
**
*
*
nomo1+/+
nomo1-/-
delayed development
pericardium edema
tail bending
yolk malformation
H
nomo1+/+
nomo1-/-
I
body length
nomo+/+
nomo-/-
the body length (μm)
15000
10000
5000
0
48hpf
14dpf
2mpf

Figure EV2. Morphological analysis of WT and *nomo1* mutant zebrafish during developmental stages.

(**A–E**) The morphology of WT and *nomo1* mutant zebrafish at 24 hpf (**A**), 36 hpf (**B**), 48 hpf (**C**), 14 dpf (**D**) and 2 mpf (**E**). (**F**) survival rate of $nomo1^{+/+}$ and $nomo1^{-/-}$ from 0 day to 60 days (biological replicates, $N = 48$). (**G**) Abnormal morphology of $nomo1^{+/+}$ and $nomo1^{-/-}$ at 24 dpf including developmental delay, tail bending, pericardium edema and yolk malformation (biological replicates, $N = 3$). (**H**) Alcian Blue and Alizarin Red staining showed Skeletal and lower jaw development of $nomo1^{+/+}$ and $nomo1^{-/-}$ at 7dpf. (**I**) The body length of $nomo1^{+/+}$ and $nomo1^{-/-}$ zebrafish at 48 hpf, 14 dpf and 2 mpf (biological replicates, $N = 36$). Data information: Data are analyzed using unpaired t test and shown as the mean ± SEM. $^{*}P < 0.05$, $^{**}P < 0.01$.

© The Author(s)

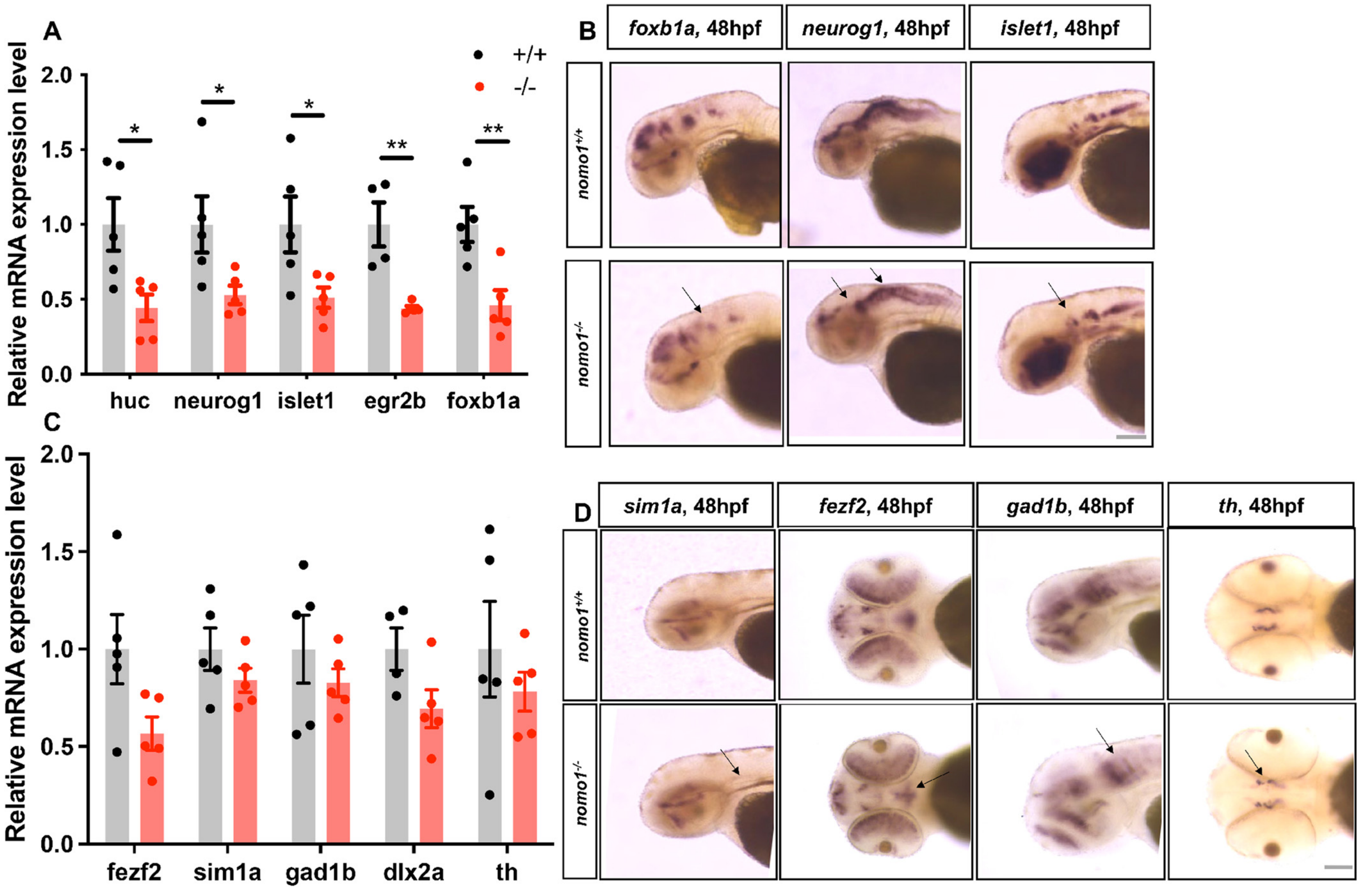

Figure EV3. Expression analysis of neurodevelopment related genes in WT and Nomo1 mutant zebrafish.

(**A–D**) (**A**, **C**) The expression level of neurological genes in brain of 48-hpf wt and *nomo1*$^{-/-}$ (biological replicates, $N = 5$). (**B**, **D**) Expression of neurological genes were detected using WISH, arrow heads indicate the expression were inhibited. Data are analyzed using unpaired *t* test and presented as the means ± SEM. *$P < 0.05$ and **$P < 0.01$. Scale bar = 100 μm.

 © The Author(s)

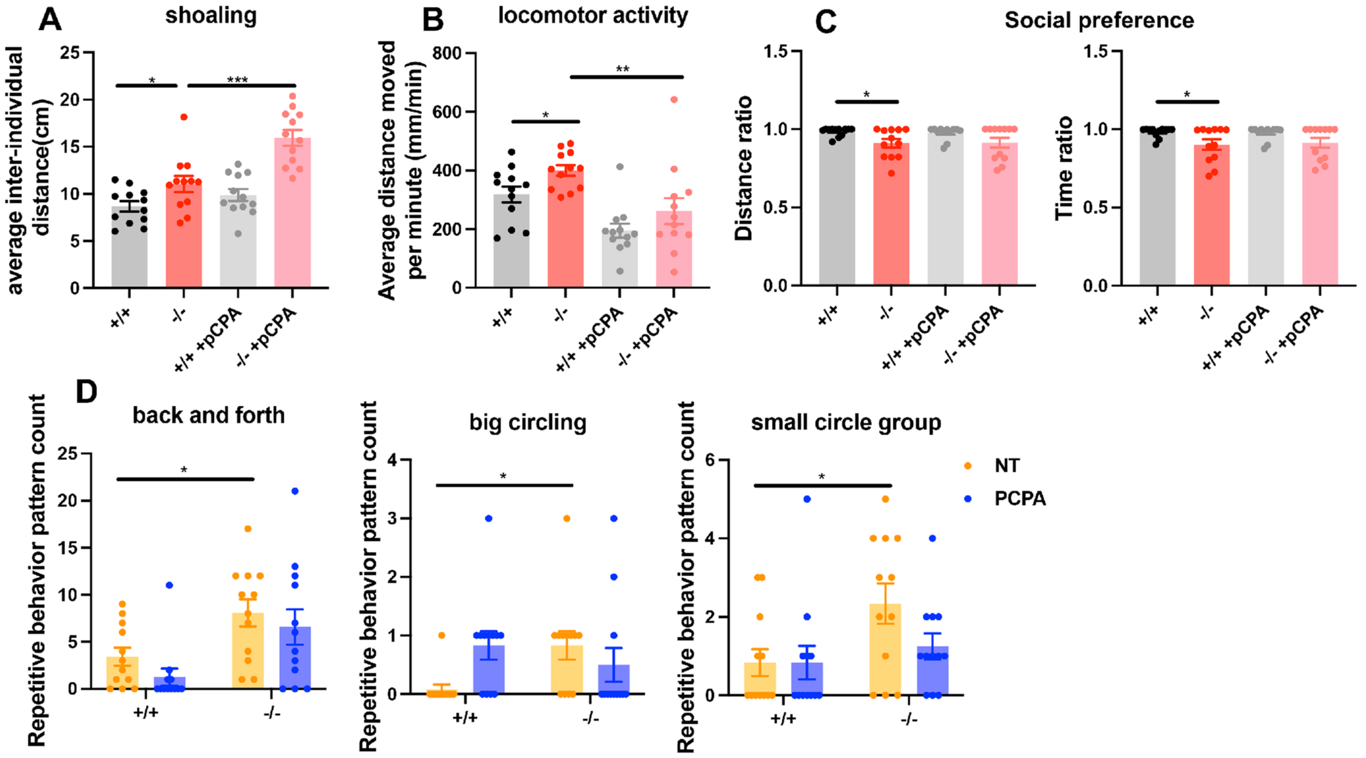

Figure EV4. pCPA treatment rescued the overactive locomotion and exacerbates social deficits of *nomo1*$^{-/-}$.

(A–D) Shoaling behavior (A) (biological replicates, $N = 12$) locomotor activity (B) (biological replicates, $N = 12$), social preference (C) (biological replicates, $N = 12$) and three kinds of repetitive behaviors (D) (biological replicates, $N = 12$) of WT, mutant and pCPA treated zebrafish. NT no treatment. Data are analyzed using unpaired *t* test and presented as the means ± SEM, $^{*}P < 0.05$, $^{**}P < 0.01$, $^{***}P < 0.001$.
